# The participation of tumor residing pericytes in oral squamous cell carcinoma

**DOI:** 10.1038/s41598-023-32528-1

**Published:** 2023-04-04

**Authors:** Isabella Bittencourt do Valle, Sicília Rezende Oliveira, Janine Mayra da Silva, Gabriela Tonini Peterle, Anna Clara Gregório Có, Sebastião Silvério Sousa-Neto, Elismauro Francisco Mendonça, José Alcides Almeida de Arruda, Natália Aparecida Gomes, Gabriel da Silva, Andréia Machado Leopoldino, Soraia Macari, Alexander Birbrair, Sandra Ventorin von Zeidler, Ivana Márcia Alves Diniz, Tarcília Aparecida Silva

**Affiliations:** 1 Department of Oral Surgery, Pathology and Clinical Dentistry, School of Dentistry, Universidade Federal de Minas Gerais, Av. Antônio Carlos, 6627, room 3105, Belo Horizonte, Minas Gerais CEP: 31.270-901 Brazil; 2 Biotechnology Post-graduation Program, Centro de Ciências da Saúde, Universidade Federal do Espírito Santo, Vitória, Espírito Santo Brazil; 3 Department of Stomatology (Oral Pathology), School of Dentistry, Universidade Federal de Goiás, Goiânia, Goiás Brazil; 4 Department of Restorative Dentistry, School of Dentistry, Universidade Federal de Minas Gerais, Belo Horizonte, Minas Gerais Brazil; 5 Department of Clinical Analysis, Toxicology and Food Sciences, School of Pharmaceutical Sciences of Ribeirão Preto, Universidade de São Paulo, São Paulo, Brazil; 6 Department of Pathology, Institute of Biological Sciences, Universidade Federal de Minas Gerais, Belo Horizonte, Minas Gerais Brazil

**Keywords:** Cancer, Oral cancer

## Abstract

Pericytes are perivascular cells related to vessel structure and angiogenesis that can interact with neoplastic cells, interfering with cancer progression and outcomes. This study focused on the characterization of pericytes in oral squamous cell carcinoma (OSCC) using clinical samples and a transgenic mouse model of oral carcinogenesis. Nestin^-^/NG2^+^ (type-1) and nestin^+^/NG2^+^ (type-2) pericytes were analyzed by direct fluorescence after induction of oral carcinogenesis (4-nitroquinoline-1-oxide). Gene expression of neuron glial antigen-2 (NG2), platelet-derived growth factor receptor beta (PDGFR-β), and cluster of differentiation 31 (CD31) was examined in human OSCC tissues. The protein expression of von Willebrand factor and NG2 was assessed in oral leukoplakia (i.e., oral potentially malignant disorders) and OSCC samples. Additionally, clinicopathological aspects and survival data were correlated and validated by bioinformatics using The Cancer Genome Atlas (TCGA). Induction of carcinogenesis in mice produced an increase in both NG2^+^ pericyte subsets. In human OSCC, advanced-stage tumors showed a significant reduction in CD31 mRNA and von Willebrand factor-positive vessels. Low PDGFR-β expression was related to a shorter disease-free survival time, while NG2 mRNA overexpression was associated with a reduction in overall survival, consistent with the TCGA data. Herein, oral carcinogenesis resulted in an increase in NG2^+^ pericytes, which negatively affected survival outcomes.

## Introduction

Oral squamous cell carcinoma (OSCC) is a highly prevalent cancer worldwide, with over 177,000 deaths in 2020^1^. Despite concerns and efforts to reduce exposure to risk factors such as tobacco and alcohol and to decrease the delay in diagnosis, the incidence of OSCC is expected to rise and is anticipated to increase by over 40% by 2040^1^.

Tumor progression and dissemination is intrinsically associated with the peritumoral space and the tumor microenvironment (TME)^2–4^. Since solid tumors require the presence of new vasculature for growth and metastasis, many current cancer therapies are designed to target tumor vessels^5–8^. Angiogenesis comprises the migration and proliferation of endothelial cells and the stabilization of newly formed vessels, which are dependent on pericytes^9,10^.

Type-2 pericytes (nestin^+^/NG2^+^) are mainly recruited during tumoral angiogenesis, while type-1 pericytes (nestin^-^/NG2^+^) are essentially related to the tissue damage response^11^. The distribution of pericytes within the TME is often anomalous, ranging from high to little/absent coverage and thus reflecting dissimilar levels of vascular bed maturation operating on different types of neoplasms and even on individual tumors^12–15^. Additionally, research on angiotropism has demonstrated that tumor cells themselves may reside in a perivascular location. Neoplastic angiotropic cells compete with and imitate pericytes along the abluminal vascular surfaces in melanoma models. Tumor cells can migrate using these various migratory strategies without entering the vascular system. In addition, they make it possible to stabilize the newly formed vasculature or the vascular basement membrane matrix, a function that is typically carried out by pericytes^16,17^.

The distinct properties of pericytes and their ability to interact with neoplastic cells in the TME make them potential disease progression modifiers^18–21^. Accordingly, pericytes are directly or indirectly related to tumor size and spread and to resistance to antitumor therapy in several types of cancers, such as mammary carcinoma, melanoma, renal cell carcinoma, and glioblastomas^13,22,23^.

Although previous studies have shown changes in the population of pericytes, as well as a reduction in the number of pericytes in OSCC^24–26^, the *bona fide* participation of pericytes during the development of OSCC and its significance in terms of clinicopathological aspects, disease progression, and prognosis is not yet defined. Thus, the purpose of the present study was to characterize the population of pericytes in OSCC and correlate it with the outcomes of the disease. Understanding the potential role of pericytes in OSCC might contribute to the improvement of antiangiogenic therapy strategies.

## Materials and methods

### Animals

Initially, for comparative studies, four-week-old female wild-type (WT) C57BL/6 mice (total: n = 12; n = 6 per group) were obtained from the Animal House of Universidade Federal de Minas Gerais (UFMG). Additional four-week-old male/female nestin-GFP/NG2-DsRed (C57BL/6 genetic background) transgenic mice (total: n = 12; n = 6 per group) were obtained as previously described^11,27,28^. The colonies of double-transgenic mice present simultaneous endogenous fluorescence for nestin (neural stem cell protein) and NG2 (neuron glial antigen-2 or chondroitin sulphate proteoglycan 4—CSPG4)^29–31^. The nestin positive cells are labeled by GFP (green fluorescent protein) and NG2 positive cells are labeled by DsRed (red fluorescent protein)^29–31^.

The animal procedures and handling were performed in accordance with the Ethics Committee for the Animal Care and Use at UFMG (#412/2018) and ARRIVE guidelines. All mice were acclimatized for two weeks under controlled conditions, i.e., 12:12 h light‐dark cycle, standard diet, and water ad libitum. Animals weighing 15 to 20 g were randomly assigned to the study groups and kept in microisolators for 28 weeks. The animals were euthanized with an anesthetic overdose (300 mg/kg of ketamine and 30 mg/kg of xylazine; i.p.) and the tongue and palate mucosa were collected. After euthanasia, the mouse tongues were photographed for representative macroscopic observations.

#### Oral carcinogenesis induction

Mice were treated with the chemical carcinogen 4-nitroquinoline-1-oxide (4NQO), as previously described^32^. Briefly, 4NQO (Sigma-Aldrich, Missouri, USA) was dissolved in ethylene glycol (Sigma-Aldrich) and administered in drinking water (50 µg/mL) for 28 weeks. The control group received pure drinking water for the same period.

#### mRNA extraction and reverse transcriptase quantitative polymerase chain reaction (RT-qPCR)

RT-qPCR analysis was performed to evaluate the gene expression of CD31 (cluster of differentiation 31, also known as platelet-endothelial cell adhesion molecule-1), PDGFR-β (platelet-derived growth factor receptor beta), and NG2. WT C57BL/6 mice were euthanized and control tongues and 4NQO-induced tongues were collected for mRNA extraction. RNA was extracted from minced tongue pieces with the TRIzol™ Reagent (Invitrogen, California, USA) method. The purity of RNA was checked and quantified with a NanoDrop spectrophotometer (Thermo Fisher, Massachusetts, USA). The cDNA of each sample was obtained from 1000 ng of intact and impurity-free RNA, using the Deoxyribonuclease I (DNase I)—Amplification Grade kit (Sigma-Aldrich) and High-Capacity cDNA Reverse Transcription (Applied Biosystems, Massachusetts, USA).

The RT-qPCR assay was performed using the SYBR Green PCR Master Mix Systems (Applied Biosystems) of Applied Biosystems™ 7500 Real-Time PCR. The relative gene expression of CD31, PDGFR-β and NG2 was determined by the 2^−(ΔΔCt)^ method and normalized with the housekeeping gene glyceraldehyde 3-phosphate dehydrogenase (GAPDH)^33^. The nucleotide sequences designed for the studied genes are presented in Supplementary Table 1.

#### Characterization of nestin^+^ and NG2^+^ cells

Samples of tongue and palate mucosa were fixed in 4% paraformaldehyde (PFA) for 48 h and cryoprotected in 30% sucrose before being embedded in optimum cutting temperature (OCT) medium (Tissue-Tek, Sakura Finetek, Osaka, Japan). Next, 15 μm thick sections were obtained by cryostat cutting (Leica, Wetzlar, Germany). The sections were counterstained with 4′,6-diamidino-2-phenylindole (DAPI) (Abcam, Cambridge, UK) in mounting medium and analyzed under a ZEISS Axioscope 5 fluorescence microscope (ZEISS, Oberkochen, Germany). One hemi-tongue sample from each group was obtained for the detection of images stained with hematoxylin and eosin (H&E). Representative images at × 20 magnification were obtained with a light microscope (Axioskop 40 Zeiss, Göttingen, Lower Saxony, Germany).

Representative images were obtained from autofluorescent tissue histological slides at × 50, × 100 and × 200 magnification. Labeled cells were counted in 10 consecutive fields at × 200 magnification. Each field was photographed with a laser at 405 nm (DAPI—blue), 488 nm (GFP—green) and 594 nm (DsRED—red) and the images were processed and combined into a merged image. All cell quantification steps were performed by a previously calibrated examiner (I.B.V.) in the merge-imaging component using the Fiji software cell count plugin (NIH, Bethesda, USA).

Staining was considered to be positive only when co-localized with DAPI. The raw nestin^+^ cells (green), NG2^+^ cells (red) and nestin^+^/NG2^+^ (yellow) cell counts were normalized by a 1 mm^2^ area. In all tissues evaluated, cell quantification was limited to the lamina propria, immediately below the epithelium. In tumor areas, 10 consecutive fields were also quantified in the peritumoral and intratumoral regions.

### Patient samples

The Research Ethics Committee of UFMG (#3.903.442/2020) approved the present study. Patient anonymity was guaranteed according to the Helsinki Declaration and informed consent was obtained from all participants. For immunohistochemical (IHC) analysis, the study included paraffin-embedded samples of oral leukoplakia (i.e., oral potentially malignant disorders) (n = 20; 26.6% of these cases were mild dysplasia and 73.4% were moderate dysplasia), as well as OSCC samples (n = 62). For RT-qPCR, 36 OSCC fresh tissue samples were evaluated. Inclusion criteria were surgical specimens from patients with a conclusive diagnosis of OSCC. Exclusion criteria were patients with previous antineoplastic therapy, incomplete medical records, and cases with positive surgical margins. Patients were followed-up for up to 60 months and all data regarding clinicopathological aspects were obtained through interviews or active search of medical records.

#### IHC method and quantification

The slides were dewaxed in xylene and rehydrated. For antigen retrieval, the slides were immersed in EDTA, pH 8.0, at 97 °C for 25 min. Blocking steps started with avidin and biotin blocking, followed by endogenous peroxidase (3% peroxide hydrogen) neutralization and protein blocking with 1% bovine serum albumin (BSA). The slides were incubated with 1:200 rabbit anti-NG2 polyclonal antibody (clone AB5320; Merck Millipore, Massachusetts, USA) and 1:100 mouse monoclonal anti-von Willebrand factor (clone F8/86; Agilent, California, USA) for two hours at room temperature. Next, the slides were incubated with peroxidase-conjugated streptavidin (LSAB2 System-HRP), revealed with 3,3'-Diaminobenzidine (DAB), counterstained with Harry’s hematoxylin (HHS80, Sigma-Aldrich), and mounted in resin non-aqueous solution (Entellan—Merck Millipore). Negative controls were obtained by omission of the primary antibody.

The number of positive cells revealed by DAB precipitation was visualized under a light microscope (Motic, Hong Kong, China). In oral leukoplakia samples, the number of NG2-positive cells in the lamina propria just below the stratified epithelium was counted in 20 consecutive fields at × 40 magnification. In the OSCC samples, NG2-labeled cells in the peritumoral and intratumoral regions were quantified in 60 consecutives fields at × 40 magnification. In addition to DAB precipitation, the NG2 pericytes were considered positive when they were closely associated with vascular structures and had flattened nuclei and elongated cytoplasm. Von Willebrand-stained vessels were quantified by measuring the highest microvascular density (h-MVD)^34^. Three different areas of vascular hotspots were selected by scanning the section at × 100 magnification. Then, three different fields were counted in each of these areas at × 200 magnification. The highest vessel count value was taken as the h-MVD. All counting procedures were performed in a blinded fashion.

#### mRNA extraction and RT-qPCR

For mRNA extraction from human tissues, 36 OSCC samples and a healthy gingiva sample (control) were used. The mRNA extraction process, cDNA conversion and RT-qPCR followed the same steps as mentioned above for animal samples. The relative gene expression of CD31, NG2, and PDGFR-β was assessed by the 2^−(ΔΔCt)^ method and normalized by the housekeeping gene β-actin^33^. The primer sequences designed are shown in Supplementary Table 2.

### Cancer genome atlas (TCGA) data

RNA-Seq expression information and the clinicopathological data of head and neck squamous cell carcinoma (HNSCC) patients from the TCGA cohort were downloaded using the UCSC Xena Functional Genomics Explorer. Next, bioinformatics analyses were implemented in RStudio (v1.3.959) to analyze the expression of the CD31, NG2 and PDGFR-β genes in 500 tumor tissue samples and 44 normal tissue samples. Gene expression data were plotted using the R ggplot2 package (v3.3.2). The correlation matrix plots were generated using the R Corrplot package (v 0.84). Comparison between two groups was performed using the Wilcoxon–Mann–Whitney test, and comparison among three or more groups was performed by the Kruskal–Wallis test. Statistical significance was considered when *p* < 0.05. The survival curves of HNSCC were drawn using the Kaplan–Meier Plotter online tool (http://kmplot.com/analysis/).

### Statistical analysis

The GraphPad Prism software version 8.0.0 for Windows (GraphPad Software, California, USA) was used for statistical analysis. Initially, descriptive analysis was performed to explore the data. After carrying out the Shapiro–Wilk and D'Agostino‐Pearson normality tests, statistically significant differences between groups were calculated by the unpaired T-test or one-way ANOVA followed by Tukey's or Dunnet's post hoc test. The nonparametric Kolmogorov–Smirnov and Kruskall-Wallis tests were adopted for the groups that did not follow Gaussian distribution. The Chi-Square test for independence was used to establish associations between the clinicopathological variables and the genic/protein expression, with a cut-off point based on the median expression of the samples studied. The optimal cut-off points for genes were 1.36 for CD31, 2.71 for NG2, and 2.50 for PDGFR-β. The cut-off points established for protein expression were 2.0 for NG2 and 6.0 for the von Willebrand factor.

The correlation matrix data were obtained and analyzed using Python, a high-level programming language (Python Software Foundation, https://www.python.org/). The main library used was Scipy, an open-source set of scientific tools for Python. Relative gene expression data were log2 transformed and tested for normal distribution based on the D'Agostino and Pearson test. Next, the Pearson correlation coefficient was tested to measure the linear relationship among gene expression datasets.

Survival curves were plotted with the Statistical Package for the Social Sciences (IBM Corp. Released 2011. IBM SPSS Statistics for Windows, Version 20.0. Armonk, NY: IBM Corp.) using the Kaplan–Meier model with a confidence level of 95%. For the Cox proportional model, the independent variables first composed a univariate regression model and then were selected to form a multiple regression model when *p* < 0.20. Statistical tests for particular experiments are mentioned in the figure legends. For all analyses, the level of significance was set at *p* < 0.05.

### Ethical approval and consent to participate

This study received approval from the Ethics Committee on Animal Use (#412/2018) and from the Ethics Committee on Human Research (#3.903.442/2020), both from Universidade Federal de Minas Gerais, Brazil. The study was performed in accordance with the Declaration of Helsinki.

## Results

### Increased numbers of nestin^-^/NG2^+^ and nestin^+^/NG2^+^ pericytes were observed in chemical carcinogenesis-induced tongue lesions

Clinically, mice treated with 4NQO exhibited exophytic, papillomatous, white, and attached-base lesions (Supplementary Fig. 1b). No clinical or microscopic changes were observed on the lingual surface in the animals of the control group (Supplementary Fig. 1a,c). Conspicuously, after histological observation, tongues from control mice showed a very clearly defined epithelium with a single layer of basal cells (Supplementary Fig. 1c), whereas the tongue lesions exposed to 4NQO showed invasive neoplastic epithelial cells inside the connective tissues (Supplementary Fig. 1d).

We used the 4NQO-induced carcinogenesis model to mimic human tobacco abuse and further identify changes in the vascular space, precisely in terms of the presence of pericytes (identified by nestin-GFP/NG2-Dsred) after OSCC progression. Initially, we investigated whether genes related to vasculature and pericytes were similarly regulated in normal and altered oral tissues. CD31 mRNA expression was consistent with NG2 and PDGFR-β expression, as observed in both control and 4NQO tongues (Supplementary Fig. 1e–g); however, no significant differences were observed in expression levels.

**Figure 1:**
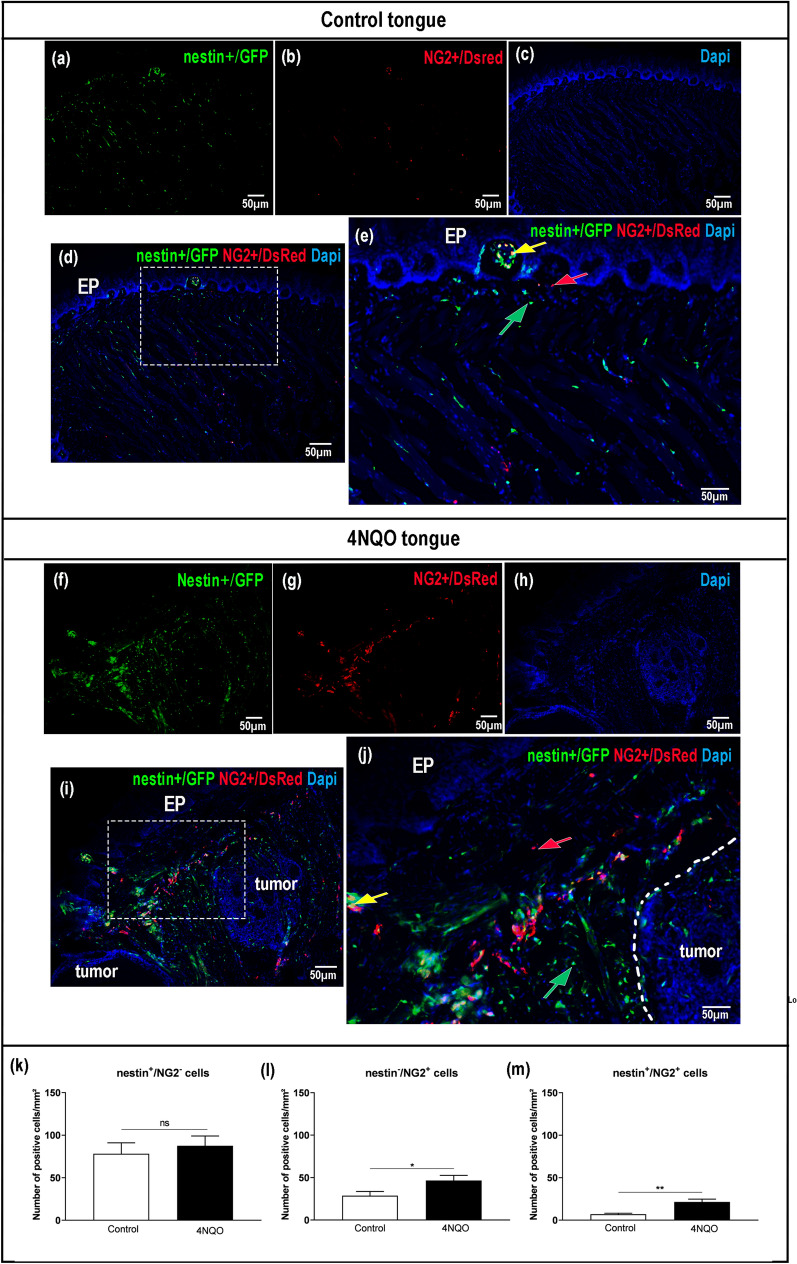
Labeled cell localization in control and 4-nitroquinoline-1-oxide (4NQO)-induced tongues. Epifluorescence of representative sections reveals nestin-GFP (green), NG2-DsRed (red), and a double-labeled population (green–red overlap). (**a–e**) Control tongue. (**a**) nestin‐GFP, (**b**) NG2‐DsRed, (**c**) 4′,6-diamidino-2-phenylindole (DAPI) split channels, and (**d, e**) composite epifluorescence image. (**f–j**) 4NQO-induced tongue. (**f**) nestin‐GFP, (**g**) NG2‐DsRed, (**h**) DAPI split channels, and (**i–j**) composite epifluorescence image. The colored arrows represent positive cells for each cell subtype evaluated. (**k–m**) Comparisons of the specific labeled cell subtypes between control and 4NQO-treated tongues are indicated in the bar graphs. Note: original magnification × 20. Scale bar: 50 μm. Statistical significance was assessed by the unpaired Student t-test. Data are shown as mean ± standard error of the mean (SEM). (*): *p* ≤ 0.05; (**): *p* ≤ 0.01. EP: epithelium and ns: nonsignificant.

Next, we sought to characterize and identify the specific tissue localization of NG2^+^ pericytes and nestin^+^ progenitor cells by choosing a transgenic model that allows the identification of distinct pericyte phenotypes. The reporter genes revealed the presence of nestin^+^/NG2^-^, nestin^-^/NG2^+^ and cells that colocalized nestin and NG2 in the evaluated oral tissues, i.e., tongue and palate mucosa (Fig. 1a–m; Supplementary Fig. 2a–o). Overall, there was a predominance of cells expressing nestin in the treated and control tongue compared to NG2 expressing cells (Fig. 1k–m). The findings in the tongue also revealed an increase in nestin^-^/NG2^+^ and nestin^+^/NG2^+^ cell types after induction of oral carcinogenesis when compared to the same healthy tissues (Fig. 1l,m). Remarkably, a population of nestin^-^/NG2^+^ and nestin^+^/NG2^+^ cells surrounding neoplastic cells was observed and these cells were also present between tumor cells (Fig. 1j).

Since the induction of oral carcinogenesis by the administration of 4NQO was not expected to produce macroscopic or microscopic changes in the palatal mucosa, we used this tissue as an internal control sample. All cells expressing nestin and NG2 were quantitatively similar in control and 4NQO-treated animals (Supplementary Fig. 2).

### Expression of endothelial cells and pericyte-related genes was correlated with the clinical characteristics and survival of patients with OSCC

**Figure 2:**
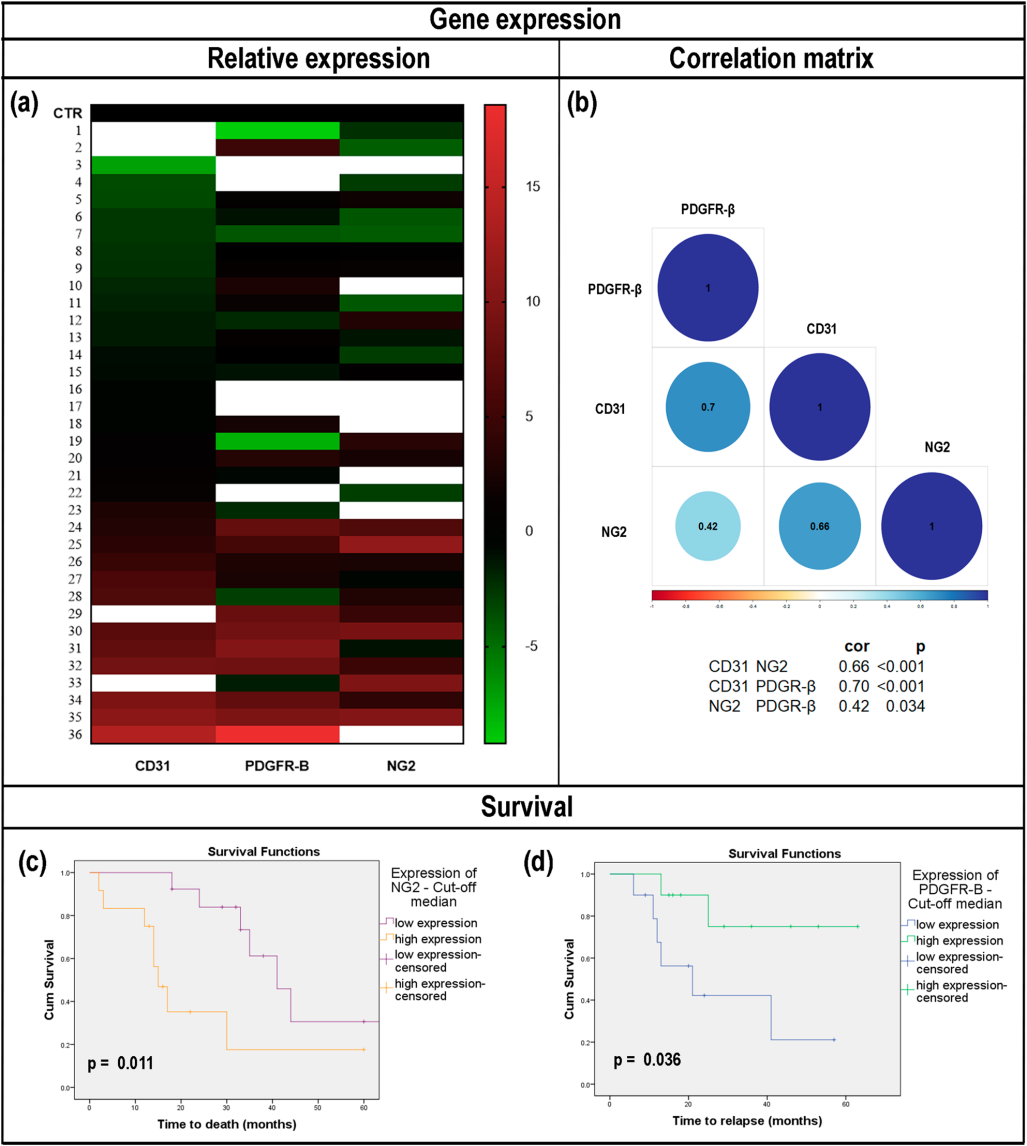
Expression of cluster of differentiation 31 (CD31), neuron glial antigen-2 (NG2), and platelet-derived growth factor receptor beta (PDGFR-β) genes in human oral squamous cell carcinoma (OSCC). (**a**) Heat map of relative expression of the studied targets (log2 transformed). Each sample is represented on a line, and the different genes analyzed are represented in the columns. The highest gene expressions are shown in red and the lowest ones in green. CTR: control (healthy gingiva). The blank spaces represent lack of gene expression for a determined sample. (**b**) Correlation matrix of Pearson's correlation for the mRNA expression of CD31, NG2, and PDGFR-β. Pairwise correlation analyses were performed for all assayed mRNAs. Blue represents a positive correlation for a given gene pair and red represents a negative correlation. CD31 and PDGFR-β showed a strong positive correlation (*r* = 0.70). There were moderate positive correlations between CD31 and NG2 (*r* = 0.66), and a low positive correlation between NG2 and PDGFR-β (*r* = 0.42). Kaplan–Meier survival curves of patients with OSCC and either high or low expression of (**c**) NG2 and (**d**) PDGFR-β. (**c**) High NG2 expression levels reveal low overall survival (OS) rates (*p* = 0.011) and (**d**) loss of PDGFR-β indicates reduced disease-free survival (DFS) (*p* = 0.036). Note: statistical significance was assessed by the log-rank test.

Thirty-six freshly isolated postoperative neoplastic samples were included in this analysis (Supplementary Table 3). The gene expression of CD31, NG2 and PDGFR-β was evaluated by RT-qPCR in tumor samples (Fig. 2a) and was found to vary among subjects, although it was possible to observe that certain expressions changed together. There was a strong positive correlation between CD31 and PDGFR-β mRNA expression (*r* = 0.70; *p* < 0.001), while there were moderate positive correlations between CD31 and NG2 (*r* = 0.66; *p* < 0.001), and a low positive correlation between NG2 and PDGFR-β (*r* = 0.42; *p* = 0.034) (Fig. 2b). Moreover, the relative expression of the three studied genes was associated with the clinicopathological features of patients with OSCC. Advanced stage tumors (stage III–IV) showed a significant reduction in CD31 and PDGFR-β expression (*p* = 0.035 and *p* = 0.020, respectively) (Supplementary Table 4).

Few environmental factors such as smoking and the concomitant habit of consuming tobacco and alcohol were found to have an impact on overall survival (OS) or disease-free survival (DFS) among patients. Individuals who smoked or who smoked and drank alcohol died earlier (*p* = 0.006 and *p* = 0.009, respectively) and showed early metastatic events when compared with individuals without such habits (*p* = 0.003 and *p* = 0.014, respectively) (Supplementary Table 5).

Regarding the impact of gene expression on survival, a high expression of NG2 mRNA was associated with a reduction in mean OS (*p* = 0.011), while a low expression of PDGFR-β was related to a shorter time of DFS (*p* = 0.036) (Fig. 2c,d; Table 1). Multiple regression applied to survival analysis (i.e., proportional hazard models) was used to estimate the role of independent variables that act multiplicatively on the risk of death and recurrence. Cox analysis indicated that high NG2 expression can promote a 7.528 times greater risk of death occurrence (HR = 7.528; 95% CI: 1.475–38.415; *p* = 0.015), while the reduction in PDGFR-β expression levels generated a 209.874-fold increase in the risk of cancer recurrence (HR = 209.874; 95% CI: 4.743–9287.552; *p* = 0.006) (Table 2).

**Table 1:** Association of cluster of differentiation 31 (CD31)^a^, neuron glial antigen-2 (NG2)^a^, and platelet-derived growth factor receptor beta (PDGFR-β)^a^ gene expression with overall survival (OS) and disease-free survival (DFS) of patients with oral squamous cell carcinoma (*n* = 36).

Gene expression	Mean OS (months)	SD	95% CI	*p* log rank	Mean DFS (months)	SD	95% CI	*p* log rank
CD31								
Above cut-off	36.100	6.516	23.323–48.877	0.983	47.533	7.283	33.260–61.807	0.555
Below cut-off	41.729	6.867	28.270–55.188		38.027	5.652	26.950–49.105	
NG2								
Above cut-off	23.260	6.343	10.828–35.693	**0.011**	53.500	8.672	36.502–70.498	0.382
Below cut-off	47.704	7.176	33.369–61.769		36.488	5.645	25.424–47.552	
PDGFR-β								
Above cut-off	41.492	7.263	27.256–55.728	0.366	52.300	6.651	15.242–41.308	**0.036**
Below cut-off	34.459	6.636	21.453–47.465		28.275	6.650	39.264–65.336	

^a^CD31: 4 samples showed no amplification; NG2: 8 samples showed no amplification; PDGFR-β: 5 samples showed no amplification.

CI, confidence interval; SD, standard deviation.

Significant values are in [bold].

**Table 2:** Cox proportional-hazards model and overall survival and disease-free survival of patients with oral squamous cell carcinoma regarding gene expression (*n* = 36).

Variables	HR (95% CI)	*p*
Overall survival		
NG2 expression		
Below cut-off	1	
Above cut-off	7.528 (1.475–38.415)	**0.015**
Age		
< 60 years	1	
≥ 60 years	0.632 (0.148–2.691)	0.534
Tumor stage		
I–II	1	
III–IV	11.815 (1.682–83.016)	**0.013**
Smoking		
No	1	
Yes	1.166 (0.095–14.338)	0.905
Smoking and alcohol consumption		
No	1	
Yes	4.011 (0.587–27.393)	0.157
Disease-free survival		
PDGFR-β expression		
Below cut-off	209.874 (4.743–9287.552)	**0.006**
Above cut-off	1	
Age		
< 60 years	1	0.276
≥ 60 years	6.270 (0.231–170.317)	
Tumor stage		
II–II	1	**0.033**
III–IV	40.153 (1.342–1201.159)	
Smoking and alcohol consumption		
No	1	0.524
Yes	2.903 (0.110–76.930)	

HR, hazard ratio; NG2, neuron glial antigen-2; PDGFR-β, platelet-derived growth factor receptor beta.

Significant values are in [bold].

### NG2-positive cells surrounded neoplastic cells in human OSCC

**Figure 3:**
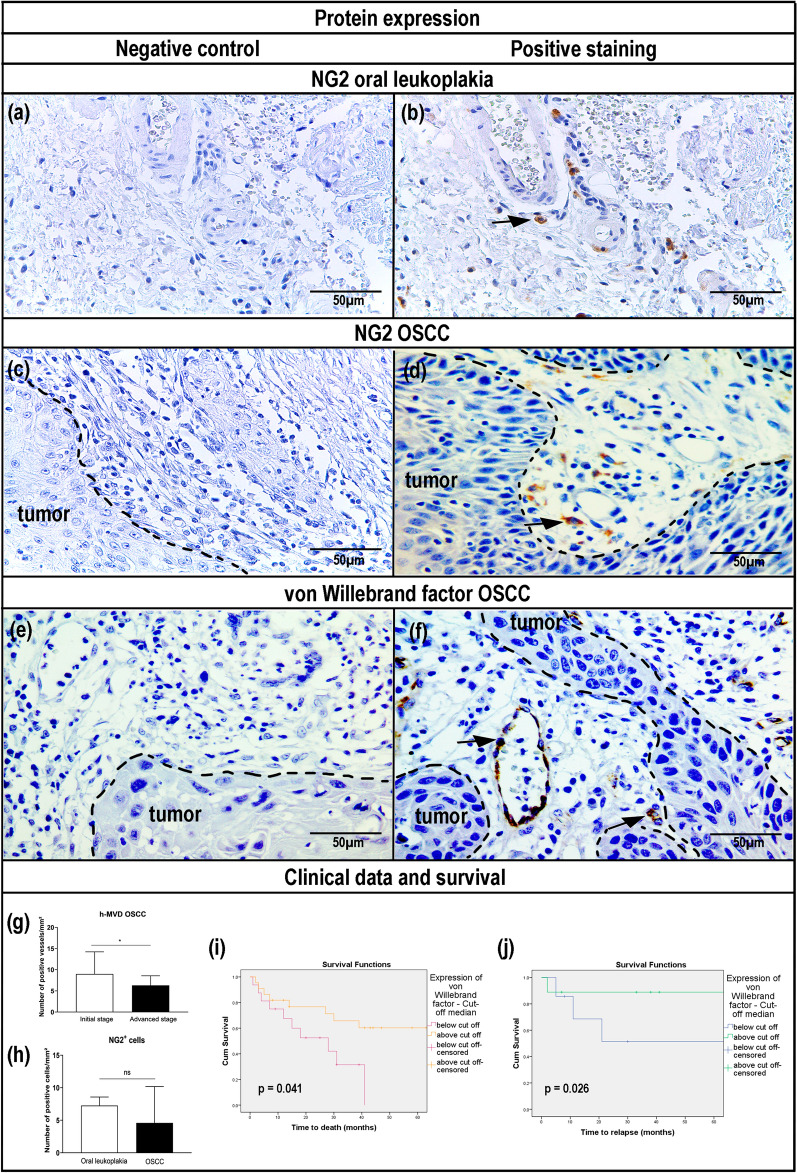
Characterization of neuron glial antigen-2 (NG2)-positive cells and von Willebrand-positive vascular structures in oral leukoplakia and oral squamous cell carcinoma (OSCC) samples. (**a, c, e**) Immunohistochemical pattern without primary antibody—negative controls. (**b**) Representative positive NG2 immunostaining in oral leukoplakia. Cell morphology indicates globular and enlarged cells with few noticeable primary branches. (**d**) Positive cells labeled with NG2 in OSCC tissue. Immunohistochemical staining reveals perivascular NG2-positive cells in peritumoral regions of primary tumor stroma. Cell morphology reveals mainly a flattened nucleus and elongating cytoplasm cells, but dissimilar shapes might be observed surrounding small vascular structures. (**f**) Vessels labeled with von Willebrand factor in OSCC demonstrate positive staining in microvessel endothelial cells or endothelial cell clusters. The bar graphs represent (**g**) NG2-positive cell quantification in oral leukoplakia versus OSCC and (**h**) highest microvascular density (h-MVD) stratified by tumor stage. Original magnification × 40. Scale bar: 50 μm. Statistical significance was assessed by the (**g**) Kolmogorov–Smirnov test, (**h**) unpaired Student t-test, and (**i, j**) log-rank test. Note: data are shown as mean ± standard deviation (SD), (*): *p* ≤ 0.05, (**): *p* ≤ 0.01, (***) *p* ≤ 0.001, ns: non-significant.

IHC expression of NG2 was analyzed in oral leukoplakia and in postoperative OSCC samples (Supplementary Table 6). In oral leukoplakia, NG2^+^ cells showed more globular and enlarged components, with few noticeable primary processes or branches (Fig. 3a,b). In the stroma of the primary OSCC and surrounding small vessels in the peritumoral regions, the cell morphology of the NG2^+^ pericytes revealed cells with a flattened nucleus and elongated cytoplasm. Some NG2^+^ cells were also observed more distantly from the perivascular regions (Fig. 3c,d). Comparison of the number of NG2^+^ cells in oral leukoplakia and in OSCC did not reveal a clear difference between the two immunostaining procedures (*p* = 0.120) (Fig. 3h).

The von Willebrand factor showed positive staining in the endothelial cells of OSCC. Any brown staining in the vascular structure or cluster of endothelial cells that was clearly separated from nearby microvessels was considered to be a countable vessel-like structure, even when there were no visible vessel lumens. In general, great microvascular density was observed in the tumor invasive front, but various tumor samples showed noticeably elevated microvessels adjacent to tumor cells (Fig. 3e,f). Regarding the higher rates of microvascular density, tumors at earlier stages (I or II) had higher vascular density than tumors at more advanced stages (III and IV) (*p* = 0.028) (Fig. 3g). The cases that showed the greatest improvement in von Willebrand h-MVD also had better OS (*p* = 0.041) and DFS period (*p* = 0.026) (Fig. 3i,j; Table 3). In a multivariate model with other potential predictors, expression of NG2 and von Willebrand proteins was not shown to imply disease survival or recurrence (Supplementary Table 7).

**Table 3:** Association between NG2 and von Willebrand factor protein expression and overall survival (OS) and disease-free survival (DFS) in patients with oral squamous cell carcinoma (*n* = 62).

Protein expression	Mean OS (months)	SD	95% CI	*p* log rank	Mean DFS (months)	SD	95% CI	*p* log rank
NG2								
Above cut-off	74.931	19.180	37.338–112.523	0.609	65.975	22.566	21.746–110.204	0.775
Below cut-off	57.971	11.996	34.459–81.482		55.911	17.951	20.727–91.096	
Von Willebrand factor								
Above cut-off	116.436	18.217	80.731–152.140	**0.041**	127.333	14.771	98.383–156.284	**0.026**
Below cut-off	23.423	4.143	15.303–31.542		55.571	20.645	15.108–96.035	

CI, confidence interval; NG2, neuron glial antigen-2; SD, standard deviation.

Significant values are in [bold].

### Expression of vascular and pericyte-related genes in a large cohort of HNSCC supported their potential involvement in cancer outcomes

**Figure 4:**
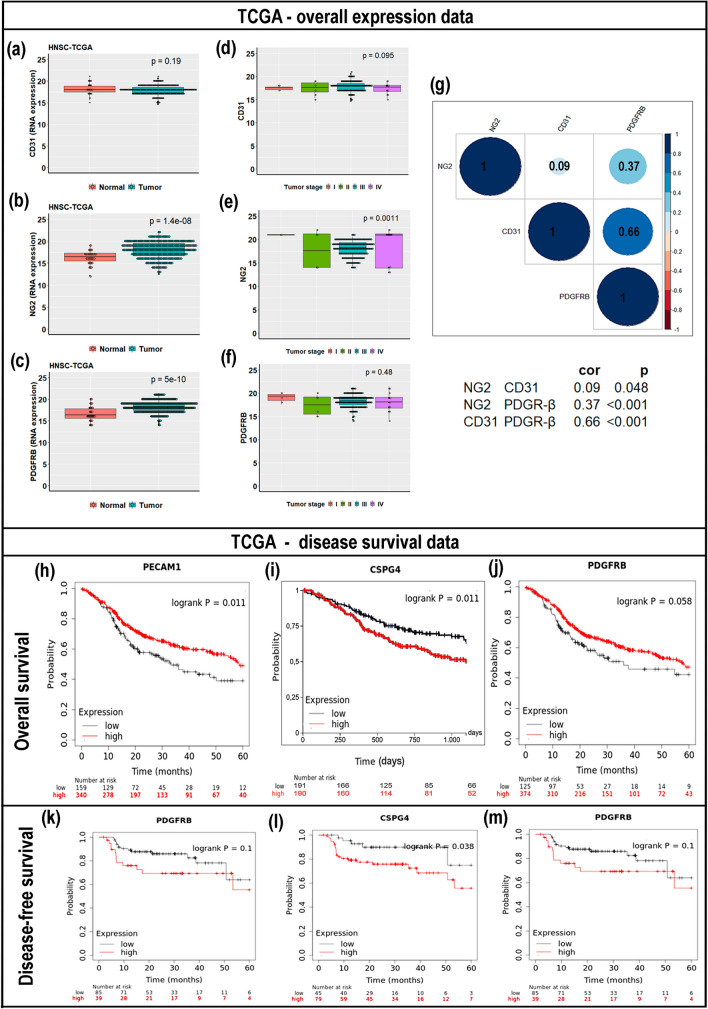
Overview of The Cancer Genome Atlas (TCGA) cohort data. Expression of (**a**) cluster of differentiation 31 (CD31), (**b**) neuron glial antigen-2 (NG2), and (**c**) platelet-derived growth factor receptor beta (PDGFR-β) mRNA in healthy versus oral squamous cell carcinoma (OSCC) patient samples. (**d–f**) Relative gene expression with respect to tumor stage (I, II, III, or IV). (**g**) Pairwise Pearson correlation matrix of the mRNA expression of CD31, NG2, and PDGFR-β. Blue indicates a positive correlation and red indicates a negative correlation. Darker colors are associated with stronger correlation coefficients. CD31 and PDGFR-β showed a moderate positive correlation (*r* = 0.66), while NG2 and PDGFR-β showed a low positive correlation (*r* = 0.37), and CD31 and NG2 presented a negligible correlation index (*r* = 0.09). Kaplan–Meier survival curves using TCGA data validate the prognostic value of genes with altered expression in OSCC. (**h**) The overall survival (OS) for CD31, (**i**) NG2 (CSPG4) and (**j**) PDGFR-β mRNA expression. (**k–m**) Disease-free survival (DFS) according to the low or high expression of the studied genes. Note: statistical significance was assessed by the log-rank test.

Regarding the TCGA data for HNSCC, we validated our findings externally. Although no difference was observed regarding the CD31 gene (Fig. 4a), patients with HNSCC exhibited high levels of NG2 and PDGFR-β in tumor samples compared to healthy controls (*p* < 0.001 for both genes) (Figs. 4b,c). The relative gene expression with respect to tumor stage (I, II, III, or IV) revealed increased NG2 expression in late stages compared to early ones (Figs. 4d–f).

There was a moderate positive correlation between the expression of CD31 and PDGFR-β (*r* = 0.66; *p* < 0.001) despite a low correlation between NG2 and PDGFR-β (*r* = 0.37; *p* < 0.001) and a negligible correlation between CD31 and NG2 (*r* = 0.09; *p* = 0.048) (Fig. 4g). Pericyte and vasculature-related genes were also associated with disease status (Fig. 4h–m). High levels of NG2 demonstrated lower OS (*p* = 0.011), while patients with high CD31 expression exhibited higher OS (*p* = 0.011) (Fig. 4h–i).

**Figure 5:**
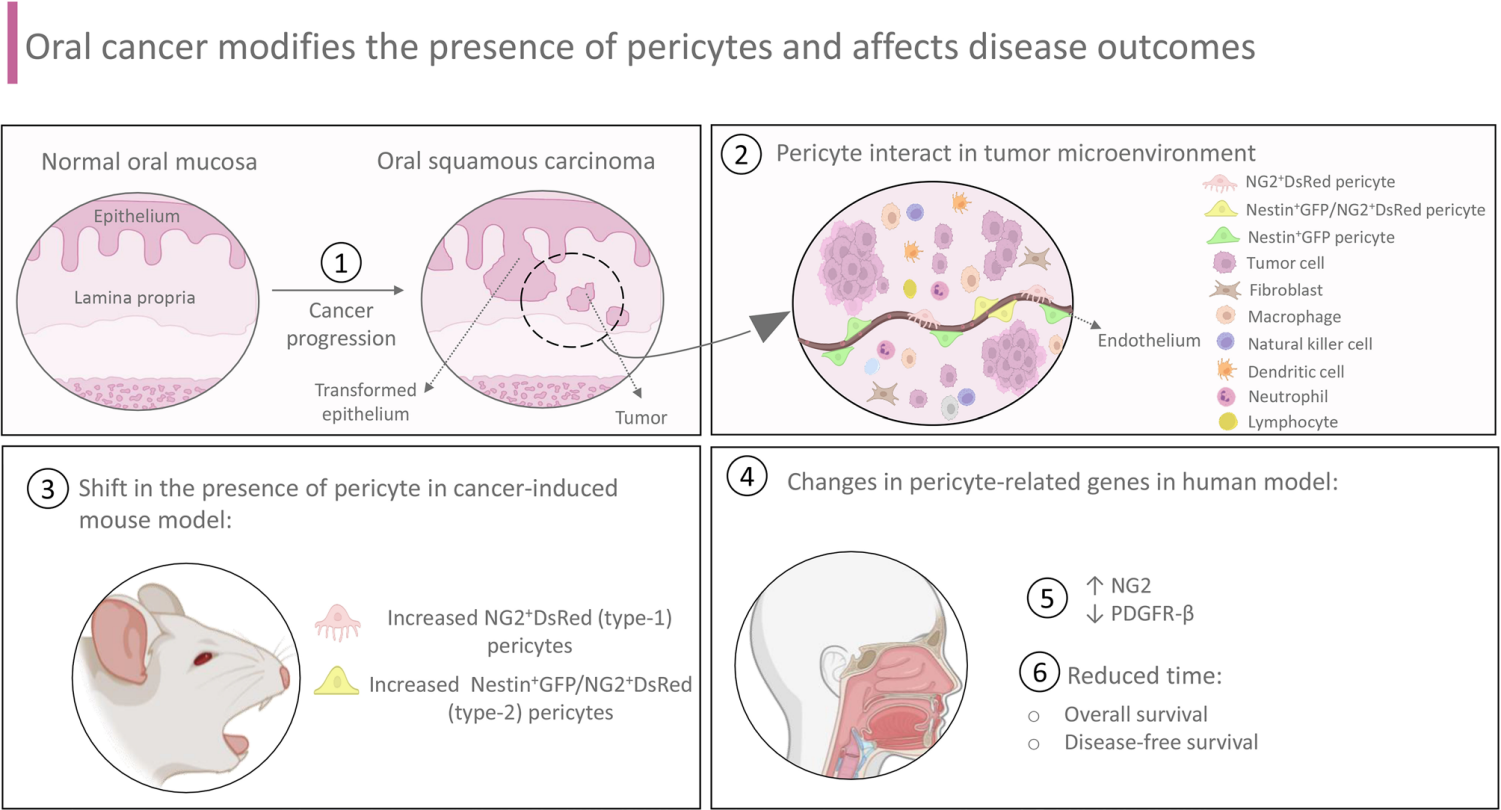
Oral cancer modifies the presence of pericytes and affects disease outcomes. (1) During the process of tumor progression and establishment of oral squamous cell carcinoma (OSCC), progressive modifications occur in the tumor microenvironment. (2) In this scenario, pericytes interact with tumor cells, other stromal cell-types and non-cellular components. (3) After oral cancer stimulation in mice, type-1 (nestin^-^/NG2^+^) and type-2 (nestin^+^/NG2^+^) pericytes were accumulated compared to healthy tissues. (4) Furthermore, in human models, alterations in pericyte-related genes influenced disease outcomes. These changes resulted in reduced patient survival time and reduced time to relapse.

A summary of our findings is illustrated in Fig. 5. A schematic representation outlines pericyte distribution changes within TME after cancer progression and spotlights the impact of this switch on OSCC outcomes.

## Discussion

Pericytes are perivascular cells involved in vessel structure and stability^35,36^. Due to their role in angiogenesis and ability to interact with TME constituents, pericytes can substantially modify cancer behavior^13^. The present study examined the relevance of pericytes in OSCC. As a whole, our analysis showed an increase in the number of nestin-GFP/NG2-DsRed pericytes in carcinogen-induced oral cancer lesions. Consistently, in human OSCC, increased NG2 gene expression was present and was implicated in worse survival time, while reduced expression of PDGFR-β was associated with a shorter time to relapse after patient treatment, suggesting an involvement in metastatic events.

It is known that OSCC has a poor prognosis, with a 5-year survival rate of around 40 to 50%^37,38^. This may be related to the limited response to multimodal treatment approaches, including antiangiogenic therapy, which remains mostly ineffective for OSCC^37,38^. From this perspective, understanding the vascular network and pericytes is fundamental for the improvement of antiangiogenic therapies. Herein, we evaluated cells that express the pericyte marker NG2 to identify a possible link between this mural cell and OSCC events. To the best of our knowledge, this is the first time that nestin^-^/NG2^+^ (type-1) and nestin^+^/NG2^+^ (type-2) pericytes have been demonstrated in oral cancer^24^.

Our investigation supported the overall expression of pericyte-related genes in normal and cancerous tongues. Furthermore, we detected a substantial increase in the number of pericytes in 4NQO-induced oral lesions, not only surrounding tumor cell islands, but also within the neoplastic tissue itself. In truth, the origin of these tumor-related pericytes is still not fully understood. In highly vascularized tumors such as glioblastomas, cancer stem cells were able to originate pericytes to sustain vascular function and ensure tumor growth via transforming growth factor beta (TGF-β) pathways. However, there was a certain range of pericytes that were re-directed from adjacent normal tissue to compose the tumoral vessels^39^.

Nestin-expressing cells were broadly detected in our control and in 4NQO-induced lesions. Previous studies have also reported the expression of nestin in healthy tissues as well as in injured tissues and tumors^29,40–43^. Basically, the expression of nestin in adult cells denotes an undifferentiated state, plasticity and increased mobility^44^. Importantly, nestin is detected in proliferating vascular endothelial cells, thus being valuable for recognition of neovascularization^45,46^. In fact, nestin expression has been detected in tumor angiogenesis in several cancer types, including glioblastoma^47^, melanoma^48^, colorectal cancer^49^ and breast cancer^50^. As a result, it is evident that nestin expression is crucial to angiogenesis^51^.

Our findings demonstrated nestin^+^ cells surrounding tumor islets/cords, probably related to the above-mentioned proliferating endothelial cells. Moreover, few malignant cells also showed noticeable nestin staining. Similarly, others have revealed nestin expression in proliferating vascular endothelial cells near a tumor and in the tumor cells themselves, including in head and neck cancer models^44,52–54^. However, we are aware of the concern that nestin expression, which occurs in a variety of cell types, does not necessarily indicate transgene activity^29^.

Nestin appears to play two roles during cancer development. First, it has been discovered that nestin is expressed in cancer stem-like cells and poorly differentiated cancer cells, suggesting that it may play a role in these cells' aggressive behavior. Second, nestin has been found to be involved in tumor angiogenesis, indicating that it may aid tumor growth^55^. Yet, the mechanisms by which nestin expression in angiogenesis occurs remain undefined^51^.

Although it has long been thought that tumor vessels fail to recruit mural cells, based on their disorganized display, chaotic vasculature and excessive ramification and leaking^19,56^, our results and those of others indicate that pericytes are, at least to some extent, present along tumor vascular tubes^19,20,57^. When evaluating the gene expression related to pericytes, using the markers NG2 and PDGFR-β together with the endothelial marker CD31, we found different patterns of expression in human neoplastic samples. Accordingly, an in vivo model of mammary tumor progression revealed that PDGFR-β pericytes were preponderant in the early stage of the tumor. Later, NG2-positive pericytes increased as the tumor grew, resulting in greater NG2/PDGFR-β overlap. However, this may illustrate how tumors at different stages, such as those investigated here, have a distinct and dynamic pericyte presence^58^.

Our analyses of TCGA data are in agreement with literature reports of a restricted distribution of NG2 in normal tissues. Moreover, we recognize that NG2 gene expression in cancer might comprise malignant cells, as well as activated pericytes and few other cells such as macrophages activated at inflammatory sites or keratinocyte progenitors in the skin^59^.

Given the different origins, there is no unique molecular marker that can be used to identify all pericyte subsets^9^. Research focused on vascular biology has considered that NG2 is a suitable marker for pericyte identification since its expression seems to be restricted to perivascular arteriolar and capillary cells during vasculogenesis/angiogenesis^10,60–62^. As this study focused on angiogenesis events, a subpopulation of venular pericytes that are negative for NG2 was not demonstrated. Furthermore, although already endorsed by other studies^11,28^, our investigation was also constrained by the fact that immunolabeling was not used to confirm the presence of nestin/NG2 with GFP/DsRed expression, and by our inability to co-localize it with vascular walls.

Despite early reports that have associated NG2 overexpression with melanomas, more recently, increased expression of NG2 has been identified in some other types of cancer such as acute myeloid leukemia, renal cell carcinomas, pancreatic carcinomas, and triple-negative breast carcinomas^59–65^. In addition, overexpression of NG2 in gliomas and triple-negative breast carcinomas has been associated with a poor prognosis, treatment resistance, and disease recurrence^37,65,66^. As also indicated by our survey and TCGA data, Warta et al.^67^ reported that NG2 is significantly overexpressed in HNSCC cancer cells. Its high expression has also been correlated with worse prognosis^67^. This NG2 overexpression is believed to contribute to cancer growth and progression by the promotion of angiogenesis; however, it remains unclear whether NG2 has a distinct function in tumor initiation, or its expression only accumulates in tumors as a secondary event^59^. Nonetheless, it has been proposed that the upregulated expression of NG2 may refer to events of hypomethylation on the gene promoter region^67^.

Another possibility is that NG2 directly facilitates communication between vascular endothelial cells and pericytes. The fact that the extracellular domain of NG2 can be shed from the cell surface by proteolysis both in vitro and in vivo adds to the appeal of this concept^68,69^. As a result, secretion of soluble NG2 by pericytes may promote recruitment of endothelial cells to neovascularization sites, stimulating the motility and morphogenesis of nearby endothelial cells^68^.

Regarding PDGFR-β, the present data indicate that its reduction occurred in advanced stage tumors, suggesting that this decrease has a role in metastatic events. In a study of breast cancer tumor transcriptomes in an extensive cohort, low PDGFR-β expression was also associated with poor recurrence-free survival^58^. Indeed, PDGF-β signaling regulates pericyte recruitment during vasculogenesis. Hyperactivation of this pathway can upturn pericyte recruitment, improving vascular stability and perfusion, which favors tumor expansion. Conversely, reduced recruitment of pericytes worsens the vessel structure, which becomes porous and permeable, facilitating tumor cell extravasation and metastatic events^13^.

In cancer models, the contribution of CD31 and von Willebrand expression to cancer development is still controversial. In particular, this may be due to the heterogeneity of patient cohorts regarding early-stage or late-stage disease. A high number of CD31-positive vessels was correlated with low T-stage and negative N-stage in laryngeal squamous cell carcinoma in a previous study^70^. Our data provided corroborating evidence by IHC that early-stage tumors had higher vascular density in the evaluated OSCC, with consequently improved OS and DFS. Recent literature has shown that, when hypoxic events are attenuated by HIF-1α, knockdown mouse models produce smaller and less hypoxic tumors^71^. These data support the notion that rapidly growing tumor cells respond to a hypoxic microenvironment with nonfunctional angiogenesis, which may represent decreased positivity for CD31/von Willebrand^71^.

In summary, our findings support altered pericyte (nestin-GPF/NG2-DsRed) presence after healthy tissue progression to OSCC, with pericyte accumulation tending to increase after neoplastic development. There is a change in NG2 and PDGFR-β mRNA expression in human OSCC that implies a worse 5-year survival and relapse. The fewer vascular density found in advanced tumors also indicated a reduced time from diagnosis to death and a decreased recurrence-free survival in patients with OSCC. It appears that a change in the presence of pericytes is essential for disease progression and severe outcomes. Taken together, our data might subsidize further therapeutic OSCC strategies targeting pericytes.

## Supplementary Information

Supplementary Information 1.

Supplementary Information 2.

Supplementary Information 3.

Supplementary Information 4.

Supplementary Information 5.

Supplementary Information 6.

Supplementary Information 7.

Supplementary Information 8.

Supplementary Information 9.

Supplementary Information 10.

## Data Availability

All data generated or analyzed during this study are included in this published article [and its supplementary information files].
