# Supplementary material for: The participation of tumor residing pericytes in oral squamous cell carcinoma
Source: Sci Rep. 2023 Apr 4;13:5460. doi: 10.1038/s41598-023-32528-1 (PMC10073133; doi:10.1038/s41598-023-32528-1)
Supplement: Supplementary file 1 — Supplementary Information 1. [file 41598_2023_32528_MOESM1_ESM.docx]

**Supplementary figure legends**

**Supplementary Figure 1.** Characterization of the oral carcinogenesis model induced by the administration of 4-nitroquinoline-1-oxide (4NQO) and healthy control. Macroscopic aspect of the tongue dorsum in **(A)** control and **(B)** 4NQO-treated mice. Clinically, lesions were frequently found in animals after induction with 4NQO. Arrows point to exophytic lesions with white, irregular surfaces, commonly located on the posterior third of the dorsum of the tongue. **(C)** Hematoxylin and eosin (H&E) representation of control tongues with normal epithelial tissue displayed in all epithelial layers. **(D**) The 4NQO tongue showed greater cell proliferation, frequent figures of atypical mitosis, altered nucleus/cytoplasm ratio and loss of polarity in the basal cells, frequently infiltrating the underlying tissue stroma. Bar graphs indicate differential expression levels of **(E)** cluster of differentiation 31 (CD31), **(F)** neuron glial antigen-2 (NG2), and **(G)** platelet-derived growth factor receptor beta (PDGFR-β). The evaluated genes were expressed in normal and altered oral tissues, with no differences noted between groups. Note: original magnification ×20. Scale bar: 100 μm. Statistical significance was assessed by the unpaired Student t-test. Data are shown as mean ± standard error of the mean (SEM). ns: non-significant.

**Supplementary Figure 2.** Labelled cell localization in control and 4-nitroquinoline-1-oxide (4NQO)-treated palate mucosa. Epifluorescence of representative sections reveal nestin-GFP (green), NG2-DsRed (red), and a double labelled population (green-red overlap). **(A)** Representative hematoxylin and eosin (H&E) staining of control palate mucosa. **(B)** Nestin‐GFP, **(C)** NG2‐DsRed, **(D)** 4′,6-diamidino-2-phenylindole (DAPI) split channels, and (**E–F**) composite epifluorescence image. **(G)** Representative H&E staining of 4NQO-treated palate mucosa. **(H)** Nestin‐GFP, **(I)** NG2‐DsRed, **(J)** DAPI split channels, and **(K-L)** composite epifluorescence image. The colored arrows represent positive cells for each cell subtype evaluated. **(M–O)** Comparisons of the specific labelled cell subtypes between control and 4NQO-treated tongue are indicated in the bar graphs. Note: original magnification ×20. Scale bars: 20 μm and 50 μm. Statistical significance was assessed by the unpaired Student t-test. Data are shown as mean ± standard error of the mean (SEM). EP: epithelium and ns: non-significant.
