# Supplementary figures and images for: The participation of tumor residing pericytes in oral squamous cell carcinoma
Source: Sci Rep. 2023 Apr 4;13:5460. doi: 10.1038/s41598-023-32528-1 (PMC10073133; doi:10.1038/s41598-023-32528-1)

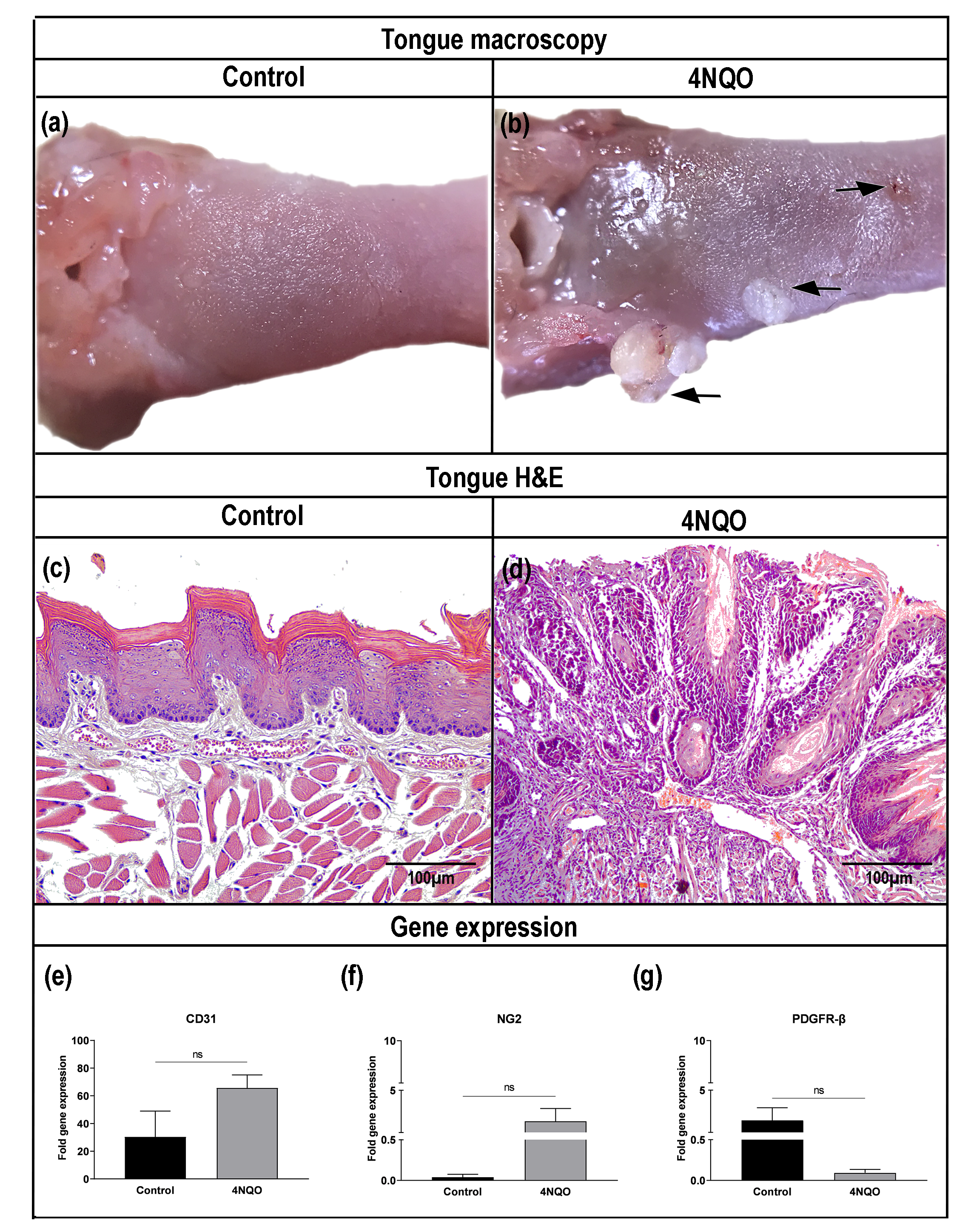

Supplement: Supplementary file 2 — Supplementary Information 2. [file 41598_2023_32528_MOESM2_ESM.tif]
