# Supplementary material for: The participation of tumor residing pericytes in oral squamous cell carcinoma
Source: Sci Rep. 2023 Apr 4;13:5460. doi: 10.1038/s41598-023-32528-1 (PMC10073133; doi:10.1038/s41598-023-32528-1)
Supplement: Supplementary file 4 — Supplementary Information 4. [file 41598_2023_32528_MOESM4_ESM.docx]

**Supplementary Table 1.** Primers designed for the amplification of cluster of differentiation 31 (CD31), neuron glial antigen-2 (NG2), platelet-derived growth factor receptor beta (PDGFR-β), and glyceraldehyde 3-phosphate dehydrogenase (GAPDH) in murine samples

| **Gene** | | **Sequence (5’-3’)** | | **Size (bp)** | |
| --- | --- | --- | --- | --- | --- |
| CD31 | F: 5’-GACTCACGCTGGTGCTCTATGC-3’ | | 114 | |  |
|  | R: 5’-TCAGTTGCTGCCCATTCATCA-3’ | |  |  |  |
| NG2 | F: 5’-CTGTTCTCACACAGAGGAGCC-3’ | | 150 | |  |
|  | R: 5’-TGGACAGACGGTCAACTTCC-3’ | |  |  |  |
| PDGFR-β | F: 5’-CCGTGGTCCCACATTCCTTG -3’ | | 131 | |  |
|  | R: 5’-TCGGATCTCATAGCGTGGCT-3’ | |  |  |  |
| GAPDH | F: 5’-AGGTCGGTGTGAACGGATTTG-3 | | 123 | |  |
|  | R: 5’-TGTAGACCATGTAGTTGAGGTCA-3’ | |  |  |  |

**Note:** F, forward; R, reverse.
