# Supplementary material for: The participation of tumor residing pericytes in oral squamous cell carcinoma
Source: Sci Rep. 2023 Apr 4;13:5460. doi: 10.1038/s41598-023-32528-1 (PMC10073133; doi:10.1038/s41598-023-32528-1)
Supplement: Supplementary file 5 — Supplementary Information 5. [file 41598_2023_32528_MOESM5_ESM.docx]

**Supplementary Table 2.** Primers designed for the amplification of cluster of differentiation 31 (CD31), neuron glial antigen-2 (NG2), platelet-derived growth factor receptor beta (PDGFR-β), and beta (β) actin in human samples

| **Gene** | **Sequence (5’-3’)** | **Size (bp)** |
| --- | --- | --- |
| CD31 | F: 5’-TCAGACGTGCAGTACACGGA-3’ | 148 |
|  | R: 5’-GGGAGCCTTCCGTTCTAGAGT-3’ |  |
| NG2 | F: 5’-GCGACTCTGGGTCTGAGGAT-3’ | 160 |
|  | R: 5’-CCTCCATCCAGGGTTCCTCT-3’ |  |
| PDGFR-β | F: 5’-TGGCCCTCAAAGGCGAG-3’ | 126 |
|  | R: 5’-GAACGAAGGTGCTGGAGACA-3’ |  |
| β-actin | F: 5’-AGGGTCTGATGGTGGGTATG-3’ | 102 |
|  | R: 5’-TGCCGTGTTCAATGGGGTAC-3’ |  |

**Note:** F, forward; R, reverse.
