# Supplementary material for: The participation of tumor residing pericytes in oral squamous cell carcinoma
Source: Sci Rep. 2023 Apr 4;13:5460. doi: 10.1038/s41598-023-32528-1 (PMC10073133; doi:10.1038/s41598-023-32528-1)
Supplement: Supplementary file 6 — Supplementary Information 6. [file 41598_2023_32528_MOESM6_ESM.docx]

**Supplementary Table 3.** Clinicopathological characteristics of patients with oral squamous cell carcinoma included in gene expression analysis (*n*=36)

| **Variables** | ***n* (%)** |
| --- | --- |
| **Anatomical location** |  |
| Tongue | 21 (58.3) |
| Floor of the mouth | 8 (22.2) |
| Others^a^ | 7 (19.5) |
| **Age** |  |
| <60 years | 13 (36.1) |
| ≥60 years | 23 (63.9) |
| **Sex** |  |
| Male | 29 (80.6) |
| Female | 7 (19.4) |
| **Tumor differentiation (*n*=34)** |  |
| Well-differentiated | 14 (38.7) |
| Moderately-differentiated | 17 (51.6) |
| Poorly-differentiated | 3 (9.7) |
| **Tumor size** |  |
| T1 | 3 (8.4) |
| T2 | 17 (47.2) |
| T3 | 3 (8.4) |
| T4 | 13 (36.0) |
| **Regional metastasis** |  |
| N0 | 23 (63.9) |
| N+ | 13 (36.1) |
| **Distant metastasis** |  |
| M0 | 34 (94.4) |
| M+ | 2 (5.6) |
| **Tumor stage** |  |
| I | 6 (16.7) |
| II | 11 (30.6) |
| III | 6 (16.7) |
| IV | 13 (36.0) |
| **Smoking (*n*=32)** |  |
| Yes | 17 (53.1) |
| No | 15 (46.9) |
| **Alcohol consumption (*n*=32)** |  |
| Yes | 19 (57.6) |
| No | 14 (42.4) |
| **Smoking and alcohol drinking (*n*=32)** |  |
| Yes | 14 (43.8) |
| No | 18 (56.2) |
| **Death occurrence (*n*=33)** |  |
| Yes | 19 (42.4) |
| No | 14 (57.6) |
| **Disease relapse (*n*=25)** |  |
| Yes | 10 (40.0) |
| No | 15 (60.0) |

**Note:** ^a^Other anatomical sites include: gingiva (*n*=5), retromolar region (*n*=1), and hard palate (*n*=1).

N0, absence of regional metastasis; N+, presence of regional metastasis; M0, absence of distant metastasis; M+, presence of distant metastasis.
