# Supplementary material for: The participation of tumor residing pericytes in oral squamous cell carcinoma
Source: Sci Rep. 2023 Apr 4;13:5460. doi: 10.1038/s41598-023-32528-1 (PMC10073133; doi:10.1038/s41598-023-32528-1)
Supplement: Supplementary file 7 — Supplementary Information 7. [file 41598_2023_32528_MOESM7_ESM.docx]

**Supplementary Table 4.** Clinicopathological characteristics of patients with oral squamous cell carcinoma (*n*=36) regarding gene expression of cluster of differentiation 31 (CD31)^a^, neuron glial antigen-2 (NG2)^a^, and platelet-derived growth factor receptor beta (PDGFR-β)^a^

| **Variables** | **CD31 expression** | | | **NG2 expression** | | | **PDGFR-β expression** | | |
| --- | --- | --- | --- | --- | --- | --- | --- | --- | --- |
|  | **Above cut-off** | **Below cut-off** | ***p***^*^ | **Above cut-off** | **Below cut-off** | ***p***^*^ | **Above cut-off** | **Below cut-off** | ***p***^*^ |
|  | ***n* (%)** | ***n* (%)** |  | ***n* (%)** | ***n* (%)** |  | ***n* (%)** | ***n* (%)** |  |
| **Anatomical location** |  |  | 0.827 |  |  | 0.743 |  |  | 0.197 |
| Tongue | 9 (28.1) | 9 (28.1) |  | 7 (25.0) | 9 (32.1) |  | 10 (32.3) | 8 (25.8) |  |
| Floor of the mouth | 3 (9.4) | 5 (15.6) |  | 4 (14.2) | 3 (10.8) |  | 4 (12.9) | 3 (9.7) |  |
| Others^b^ | 3 (9.4) | 3 (9.4) |  | 3 (10.8) | 2 (7.1) |  | 1 (3.2) | 5 (16.1) |  |
| **Age** |  |  | 0.784 |  |  | 0.115 |  |  |  |
| <60 years | 6 (18.8) | 6 (18.8) |  | 7 (25.0) | 3 (10.7) |  | 8 (25.8) | 3 (9.7) | **0.044** |
| ≥60 years | 9 (28.0) | 11 (34.4) |  | 7 (25.0) | 11 (39.3) |  | 7 (22.6) | 13 (41.9) |  |
| **Sex** |  |  |  |  |  |  |  |  |  |
| Male | 11 (34.4) | 15 (46.9) | 0.383 | 10 (35.7) | 14 (50.0) | 0.098 | 13 (41.9) | 13 (41.9) | 1.000 |
| Female | 4 (12.5) | 2 (6.2) |  | 4 (14.3) | 0 (0.0) |  | 2 (6.5) | 3 (9.7) |  |
| **Tumor differentiation (*n*=34)** |  |  |  |  |  |  |  |  |  |
| Well-differentiated | 5 (16.7) | 6 (20.0) | 0.636 | 4 (14.8) | 9 (33.3) | 0.212 | 6 (21.4) | 6 (21.4) | 0.872 |
| Moderately-differentiated | 6 (20.0) | 10 (33.3) |  | 7 (25.9) | 4 (14.9) |  | 6 (21.4) | 7 (25.0) |  |
| Poorly-differentiated | 2 (6.7) | 1 (3.3) |  | 2 (7.4) | 1 (3.7) |  | 1 (3.7) | 2 (7.1) |  |
| **Tumor size** |  |  |  |  |  |  |  |  |  |
| T1 – T2 | 10 (31.3) | 7 (21.8) | 0.149 | 6 (21.4) | 9 (32.1) | 0.256 | 10 (32.3) | 6 (19.3) | 0.104 |
| T3 – T4 | 5 (15.6) | 10 (31.3) |  | 8 (28.6) | 5 (17.9) |  | 5 (16.1) | 10 (32.3) |  |
| **Regional metastasis** |  |  |  |  |  |  |  |  |  |
| N0 | 11 (34.4) | 10 (31.2) | 0.388 | 7 (25.0) | 9 (32.1) | 0.445 | 9 (29.0) | 10 (32.2) | 0.886 |
| N+ | 4 (12.5) | 7 (21.9) |  | 7 (25.0) | 5 (17.9) |  | 6 (19.4) | 6 (19.4) |  |
| **Distant metastasis** |  |  |  |  |  |  |  |  |  |
| M0 | 15 (46.9) | 16 (50.0) | 1.000 | 13 (46.4) | 13 (46.4) | 1.000 | 14 (45.2) | 16 (51.6) | 0.484 |
| M+ | 0 (0.0) | 1 (3.1) |  | 1 (3.6) | 1 (3.6) |  | 1 (3.2) | 0 (0.0) |  |
| **Tumor stage** |  |  |  |  |  |  |  |  |  |
| I – II | 10 (31.3) | 5 (15.6) | **0.035** | 5 (17.9) | 7 (25.0) | 0.445 | 10 (32.3) | 4 (12.9) | **0.020** |
| III – IV | 5 (15.6) | 12 (37.5) |  | 9 (32.1) | 7 (25.0) |  | 5 (16.1) | 12 (38.7) |  |
| **Smoking (*n*=32)** |  |  |  |  |  |  |  |  |  |
| Yes | 7 (25.0) | 9 (32.2) | 0.743 | 9 (34.7) | 5 (19.2) | 0.249 | 8 (29.7) | 6 (22.2) | 0.332 |
| No | 6 (21.4) | 6 (21.4) |  | 5 (19.2) | 7 (26.9) |  | 5 (18.4) | 8 (29.7) |  |
| **Alcohol consumption (*n*=33)** |  |  |  |  |  |  |  |  |  |
| Yes | 9 (31.1) | 9 (31.1) | 0.812 | 9 (33.3) | 8 (29.7) | 1.000 | 10 (35.7) | 6 (21.4) | 0.127 |
| No | 5 (17.1) | 6 (20.7) |  | 5 (18.5) | 5 (18.5) |  | 4 (14.3) | 8 (28.6) |  |
| **Smoking and alcohol consumption (*n*=32)** |  |  |  |  |  |  |  |  |  |
| Yes | 5 (17.8) | 8 (28.6) | 0.431 | 7 (26.9) | 5 (19.1) | 0.671 | 6 (22.2) | 5 (18.6) | 0.581 |
| No | 8 (28.6) | 7 (25.0) |  | 7 (26.9) | 7 (26.9) |  | 7 (25.9) | 9 (33.3) |  |

**Note:** ^a^CD31: 4 samples showed no amplification; NG2: 8 samples showed no amplification; PDGFR-β: 5 samples showed no amplification.

^b^Other anatomical sites include: gingiva (*n*=5), retromolar region (*n*=1), and hard palate (*n*=1).

N0, absence of regional metastasis; N+, presence of regional metastasis; M0, absence of distant metastasis; M+, presence of distant metastasis.

^*^Pearson's chi-squared test.
