# Supplementary material for: The participation of tumor residing pericytes in oral squamous cell carcinoma
Source: Sci Rep. 2023 Apr 4;13:5460. doi: 10.1038/s41598-023-32528-1 (PMC10073133; doi:10.1038/s41598-023-32528-1)
Supplement: Supplementary file 8 — Supplementary Information 8. [file 41598_2023_32528_MOESM8_ESM.docx]

**Supplementary Table 5.** Association between the clinicopathological characteristics of individuals with oral squamous cell carcinoma and overall (OS) and disease-free survival (DFS) (*n*=36)

| **Variables** | **Mean OS (months)** | **SD** | **95% CI** | ***p* log rank** | **Mean DFS (months)** | **SD** | **95% CI** | ***p* log rank** |
| --- | --- | --- | --- | --- | --- | --- | --- | --- |
| **Anatomical location** |  |  |  |  |  |  |  |  |
| Tongue | 43.422 | 6.667 | 30.355–56.489 | 0.525 | 39.337 | 6.280 | 27.067–51.686 | 0.815 |
| Floor of the mouth | 28.429 | 5.982 | 16.704–40.153 |  | 40.600 | 6.655 | 27.556–53.644 |  |
| Others^a^ | 30.000 | 9.947 | 10.504–49.496 |  | 42.750 | 10.609 | 21.957–63.543 |  |
| **Age** |  |  |  |  |  |  |  |  |
| <60 years | 26.204 | 5.306 | 15.805–36.603 | 0.130 | 29.250 | 7.293 | 14.995–43.545 | 0.097 |
| ≥60 years | 43.475 | 6.527 | 30.683–56.268 |  | 47.987 | 5.449 | 37.307–58.667 |  |
| **Sex** |  |  |  |  |  |  |  |  |
| Male | 40.401 | 5.459 | 29.701–51.100 | 0.297 | - | - | - | - |
| Female | 23.857 | 8.854 | 06.504–41.210 |  | - | - | - |  |
| **Tumor differentiation (*n*=34)** |  |  |  |  |  |  |  |  |
| Well-differentiated | 44.703 | 8.651 | 27.746–61.660 | 0.424 | - | - | - | - |
| Moderately-differentiated | 31.079 | 5.426 | 20.445–41.713 |  | - | - | - |  |
| Poorly-differentiated |  |  |  |  |  |  |  |  |
| **Tumor size** |  |  |  |  |  |  |  |  |
| T1 – T2 | 52.188 | 6.546 | 39.358–65.017 | **0.001** | 44.313 | 5.938 | 32.675–55.950 | 0.519 |
| T3 – T4 | 21.048 | 4.814 | 11.613–30.482 |  | 31.905 | 5.474 | 21.176–42.633 |  |
| **Regional metastasis** |  |  |  |  |  |  |  |  |
| N0 | 45.567 | 6.552 | 32.726–58.408 | **0.050** | 30.857 | 5.813 | 19.463–42.251 | 0.401 |
| N+ | 25.000 | 5.732 | 13.765–36.235 |  | 45.111 | 5.771 | 33.799–56.423 |  |
| **Tumor stage** |  |  |  |  |  |  |  |  |
| I – II | 60.981 | 6.234 | 48.762–73.201 | **<0.001** | 47.000 | 5.816 | 35.601–58.399 | 0.158 |
| III – IV | 19.632 | 3.448 | 12.874–26.391 |  | 28.612 | 5.251 | 18.321–38.904 |  |
| **Smoking (*n*=32)** |  |  |  |  |  |  |  |  |
| Yes | 21.163 | 2.882 | 15.513–26.813 | **0.006** | 21.413 | 3.667 | 14.224–28.601 | **0.003** |
| No | 47.546 | 8.553 | 30.786–64.312 |  | 54.273 | 5.461 | 43.569–64.976 |  |
| **Alcohol consumption (*n*=33)** |  |  |  |  |  |  |  |  |
| Yes | 29.520 | 4.663 | 20.380–38.659 | 0.601 | 33.615 | 4.894 | 24.023–43.207 | 0.388 |
| No | 39.404 | 8.972 | 21.818–56.990 |  | 50.250 | 7.810 | 34.943–65.557 |  |
| **Smoking and alcohol consumption (*n*=32)** |  |  |  |  |  |  |  |  |
| Yes | 20.068 | 3.374 | 13.455–26.680 | **0.009** | 22.250 | 4.078 | 14.257–30.243 | **0.014** |
| No | 44.609 | 7.688 | 29.539–59.678 |  | 50.833 | 5.992 | 39.089–62.578 |  |

**Note:** ^a^Other anatomical sites include: gingiva (*n*=5), retromolar region (*n*=1), and hard palate (*n*=1).

CI, confidence interval; SD, standard deviation.
