# Supplementary material for: The participation of tumor residing pericytes in oral squamous cell carcinoma
Source: Sci Rep. 2023 Apr 4;13:5460. doi: 10.1038/s41598-023-32528-1 (PMC10073133; doi:10.1038/s41598-023-32528-1)
Supplement: Supplementary file 9 — Supplementary Information 9. [file 41598_2023_32528_MOESM9_ESM.docx]

**Supplementary Table 6.** Clinicopathological characteristics of patients with oral squamous cell carcinomas submitted to protein expression analysis (*n*=62)

| **Variables** | ***n* (%)** |
| --- | --- |
| **Anatomical location (*n*=56)** |  |
| Tongue | 37 (66.1) |
| Floor of the mouth | 8 (14.3) |
| Others^a^ | 11 (19.6) |
| **Age (*n*=56)** |  |
| <60 years | 28 (50.0) |
| ≥60 years | 28 (50.0) |
| **Sex (*n*=55)** |  |
| Male | 39 (70.9) |
| Female | 16 (29.1) |
| **Tumor differentiation (*n*=53)** |  |
| Well-differentiated | 14 (26.4) |
| Moderately-differentiated | 21 (39.6) |
| Poorly-differentiated | 18 (34.0) |
| **Tumor size (*n*=56)** |  |
| T1 | 10 (17.9) |
| T2 | 16 (28.5) |
| T3 | 15 (26.8) |
| T4 | 15 (26.8) |
| **Regional metastasis (*n*=56)** |  |
| N0 | 27 (48.2) |
| N+ | 29 (51.8) |
| **Tumor stage (*n*=56)** |  |
| I | 7 (12.5) |
| II | 13 (23.2) |
| III | 20 (35.7) |
| IV | 16 (28.6) |
| **Smoking (*n*=50)** |  |
| Yes | 44 (88.0) |
| No | 6 (12.0) |
| **Alcohol consumption (*n*=47)** |  |
| Yes | 33 (70.2) |
| No | 14 (29.8) |
| **Smoking and alcohol consumption (*n*=47)** |  |
| Yes | 31 (66.0) |
| No | 16 (34.0) |
| **Death occurrence (*n*=52)** |  |
| Yes | 28 (53.8) |
| No | 24 (46.2) |
| **Disease relapse (*n*=25)** |  |
| Yes | 11 (44.0) |
| No | 14 (56.0) |

**Note:** ^b^Other anatomical sites include: gingiva (*n*=3), retromolar region (*n*=2), alveolar ridge (*n*=1), hard palate (*n*=3), and buccal mucosa (*n*=2).

N0, absence of regional metastasis; N+, presence of regional metastasis.
