# Supplementary material for: The participation of tumor residing pericytes in oral squamous cell carcinoma
Source: Sci Rep. 2023 Apr 4;13:5460. doi: 10.1038/s41598-023-32528-1 (PMC10073133; doi:10.1038/s41598-023-32528-1)
Supplement: Supplementary file 10 — Supplementary Information 10. [file 41598_2023_32528_MOESM10_ESM.docx]

**Supplementary Table 7.** Cox proportional-hazards model, overall survival and disease-free survival of patients with oral squamous cell carcinoma in relation to protein expression (*n*=62)

|  | **Overall survival** |  |
| --- | --- | --- |
| **Variables** | **HR (95% CI)** | ***p*** |
| **von Willebrand factor** |  | 0.193 |
| Below cut-off | 2.025 (0.700–5.859) |  |
| Above cut-off | 1 |  |
| **Tumor stage** |  |  |
| I – II | 1 | 0.164 |
| III – IV | 2.606 (0.677–10.038) |  |
| **Tumor differentiation** |  |  |
| Well-differentiated | 1 |  |
| Moderately-differentiated | 1.696 (0.394–7.312) | 0.478 |
| Poorly-differentiated | 1.816 (0.435–7.589) | 0.413 |
|  | **Disease-free survival** |  |
| **Variables** | **HR (95% CI)** | ***p*** |
| **von Willebrand factor** |  |  |
| Below cut-off | 4.374 (0.117–164.166) | 0.425 |
| Above cut-off | 1 |  |
| **Age** |  |  |
| <60 years | 1 | 0.754 |
| ≥60 years | 1.790 (0.047–68.062) |  |
| **Tumor stage** |  |  |
| I – II | 1 | 0.643 |
| III – IV | 1.708 (0.178–16.415) |  |

**Note:** CI, confidence interval; HR, hazard ratio.
